# Supplementary material for: Sterols are required for the coordinated assembly of lipid droplets in developing seeds
Source: Nat Commun. 2021 Sep 22;12:5598. doi: 10.1038/s41467-021-25908-6 (PMC8458542; doi:10.1038/s41467-021-25908-6)
Supplement: Supplementary file 1 — Supplementary Information [file 41467_2021_25908_MOESM1_ESM.pdf]

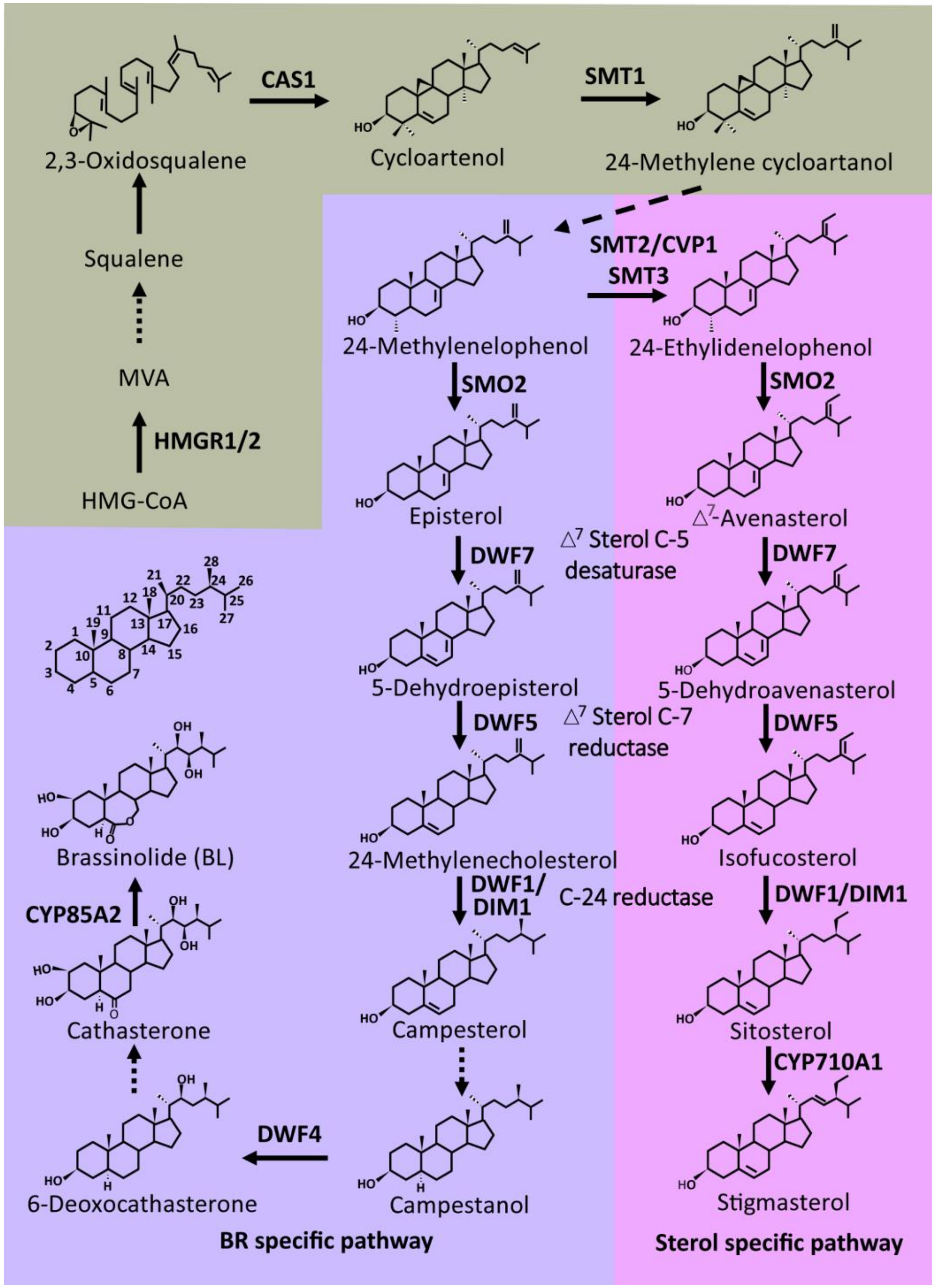

Supplementary Fig. 1 Schematic diagram of the biosynthetic pathway of plant sterols. Enzymes are shown in bold. Solid arrows and dashed arrows indicate single-step reactions and several steps, respectively. DWF4 and CYP85A2 are BR specific pathway enzymes. HMG-CoA, 3-hydroxy-3-methylglutaryl-CoA; HMGR, 3-hydroxy-3-methylglutaryl-CoA reductase; MVA, mevalonic acid; CAS1, cycloartenol synthase 1; SMT1, C-24 sterol methyltransferase 1; SMO2, C-4 sterol methyl oxidase 2; SMT2/CVP1, C-24 sterol methyltransferase 2/cotyledon vascular pattern 1; SMT3, C-24 sterol methyltransferase 3; DWF7, dwarf7; DWF5, dwarf5; DWF1/DIM1-1, dwarf1/diminuto1.

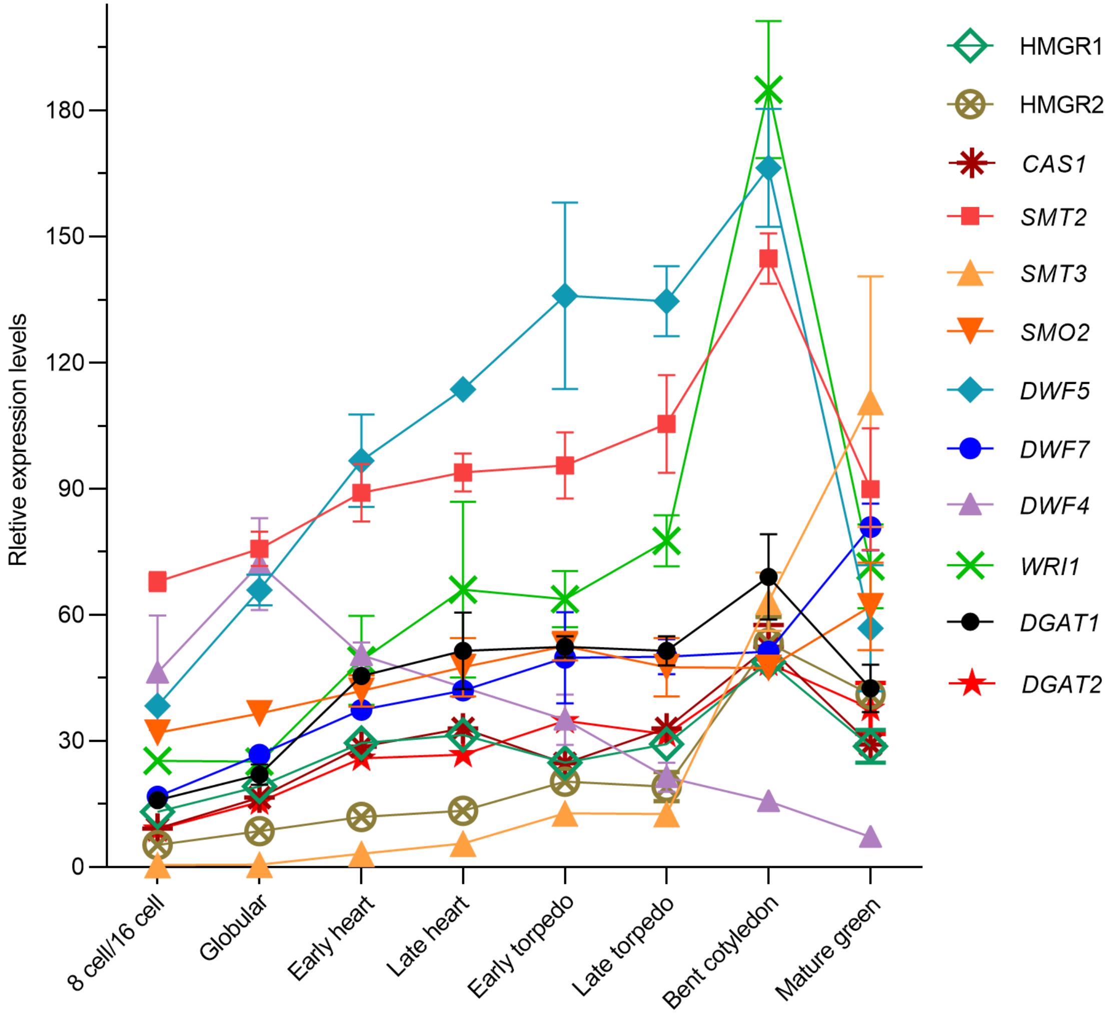

Supplementary Fig. 2 Expression levels of key sterol and TAG biosynthesis genes in developing embryos. Expression data were extracted from the Arabidopsis eFP browser. The data source of eFP browser was set to the Embryo, with absolute values for gene expression. Data are mean ± s.d. of three biological replicates.

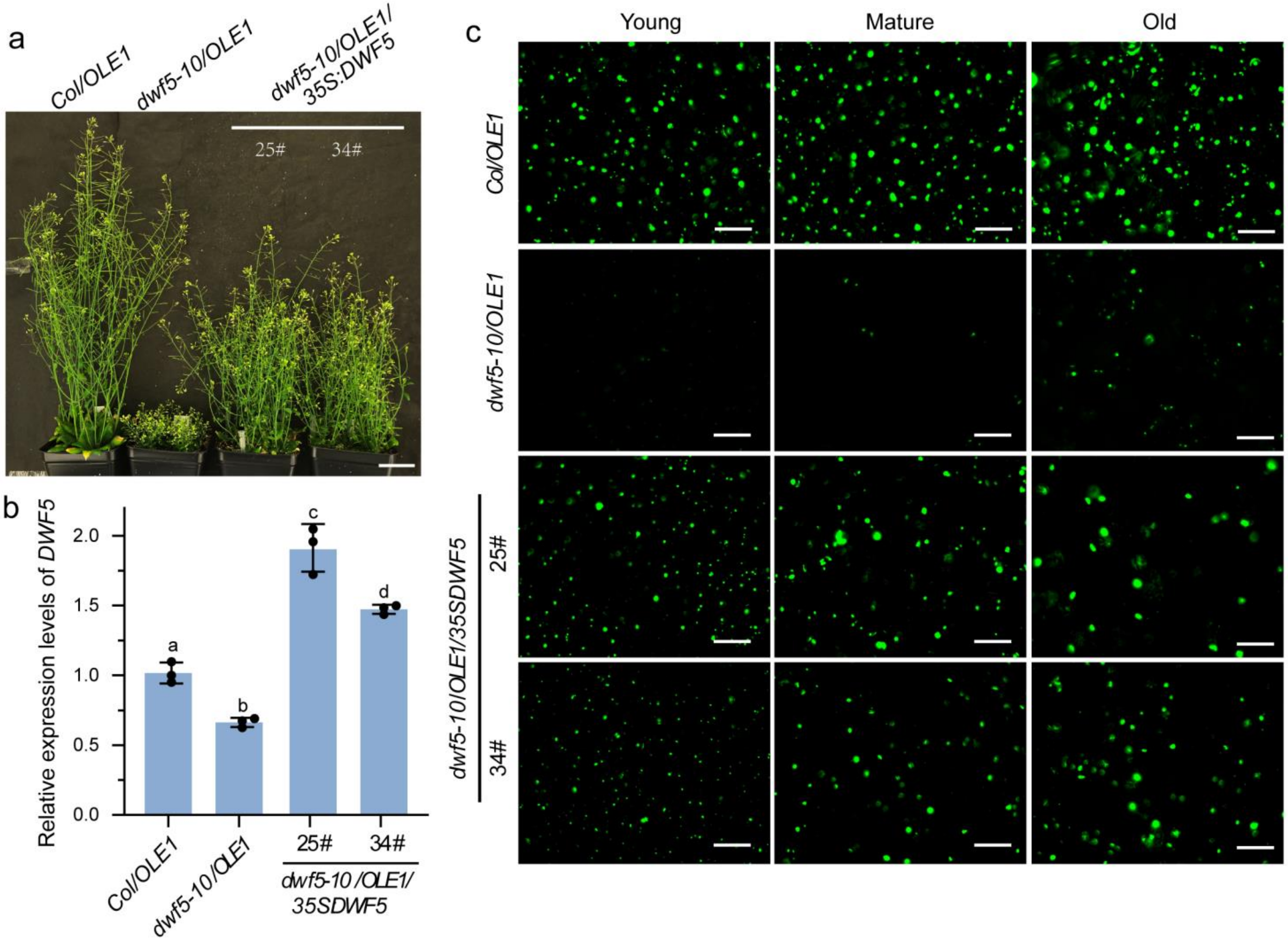

Supplementary Fig. 3 Functional complementation analysis of *dwf5-10*. **a** Growth phenotypes of 6-week-old *dwf5-10*/*OLE1/35S:DWF5* lines. Bar = 4 cm. **b** *DWF5* expression levels in *Col/OLE1*, *dwf5-10*/*OLE1*, *dwf5-10*/*OLE1*/*35S*:*DWF5* plants quantified by qRT-PCR. Data are mean ± s.d. of three biological replicates. Different letters indicate significant differences at $P < 0.05$, as determined by one-way ANOVA with Tukey's multiple comparisons test. **c** Representative images of LDs (OLE1-GFP, green) in young, mature and old leaves of 4-week-old *Col/OLE1*, *dwf5-10/OLE1*, *dwf5-10*/*OLE1*/*35S:DWF5* plants. The experiment was repeated 3 times with similar results. Bar = 50 μm.

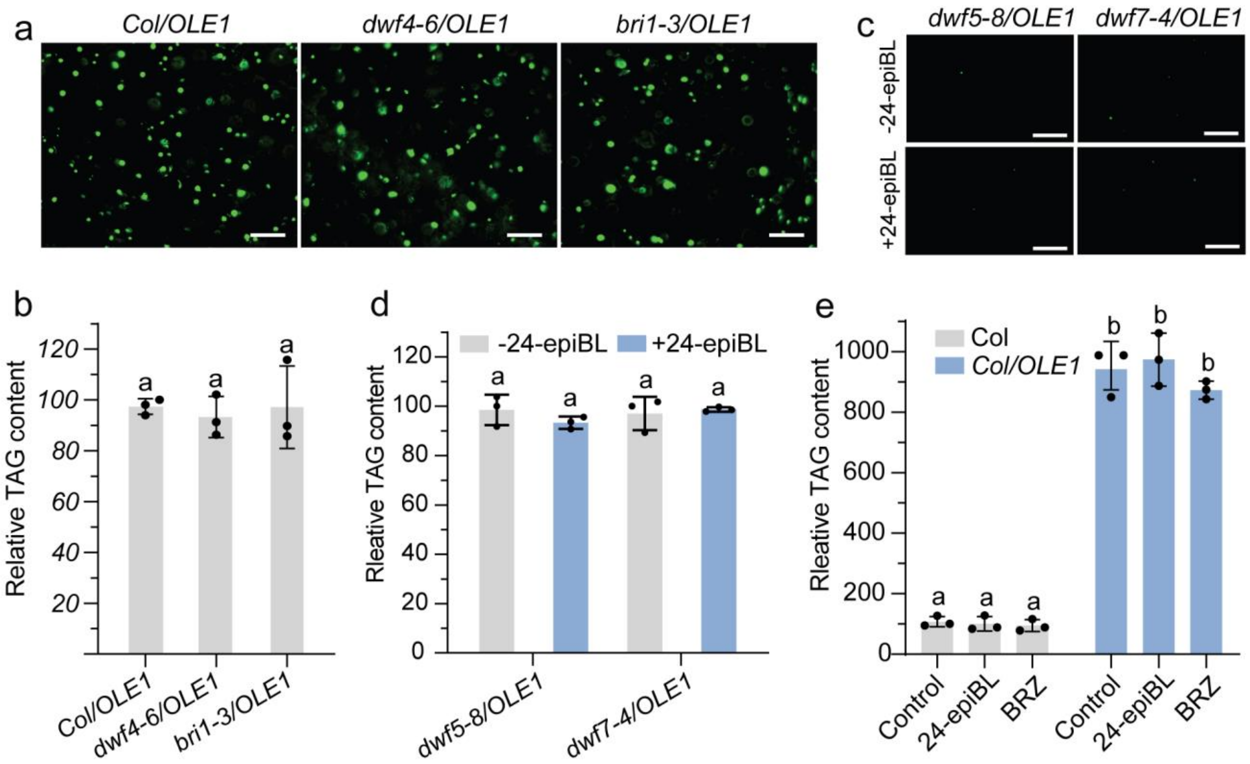

Supplementary Fig. 4 BRs and BR signaling do not affect TAG and LD accumulation in leaves. a Representative images of LDs (OLE1-GFP, green) in mature leaves of 4-week-old *Col/OLE1*, *dwf4-6*/*OLE1* and *bri1-3*/*OLE1* plants. The experiment was repeated 3 times with similar results. Bar = 50 μm. **b** Relative TAG contents in mature leaves of 4-week-old *Col/OLE1*, *dwf4-6*/*OLE1* and *bri1-3*/*OLE1* plants. **c-d** Representative images of LDs (**c**) and TAG contents (**d**) in leaves of *dwf5-8*/*OLE1* and *dwf7-4/OLE1* treated with or without 1 μM 24-epiBL. Thirteen-day-old seedlings were transferred to ½ MS medium with or without 1 μM 24-epiBL and incubated for 3 days prior to microscopic and TAG analyses. Bar = 50 μm. **e** Relative TAG contents in shoots of wide type and *Col/OLE1* plants treated with 24-epiBL and BRZ. Ten-day-old wide type and *Col/OLE1* seedlings were transferred to ½ MS medium with or without 1 μM 24-epiBL or BRZ and incubated for 3 days prior to TAG analysis. Relative TAG levels were quantified by Image J. In **b**, **d, and e,** data are mean ± s.d. of three biological replicates. Different letters indicate significant differences at $P < 0.05$, as determined by one-way ANOVA with Tukey's multiple comparisons test.

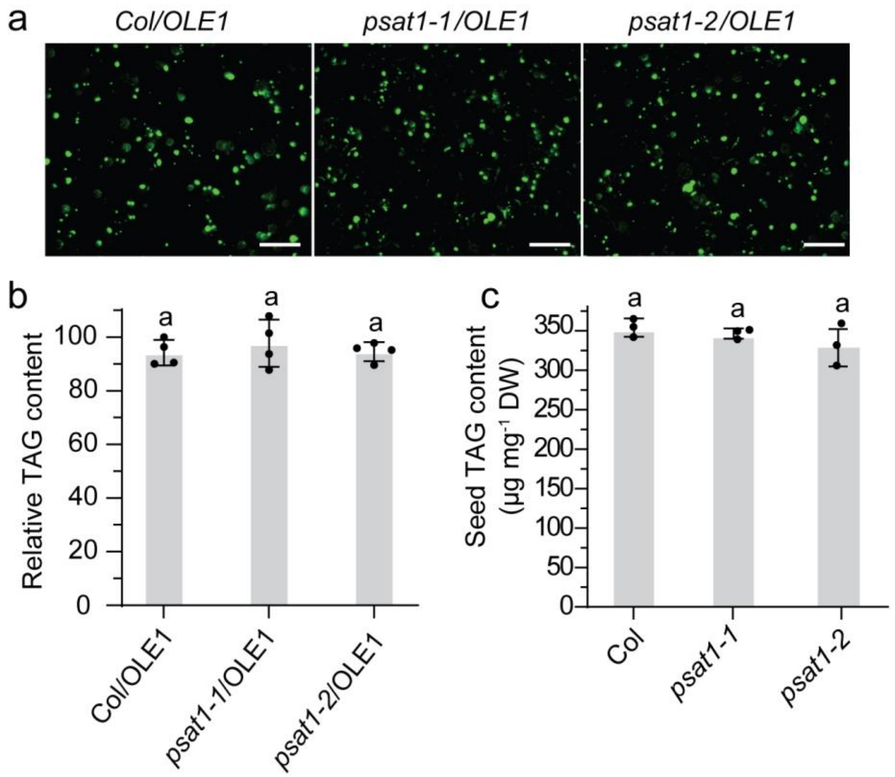

Supplementary Fig. 5 Deficiency in SEs does not affect TAG accumulation. **a** Representative images of LDs (OLE1-GFP, green) in mature leaves of 4-week-old *Col/OLE1*, *psat1-1/OLE1*, *psat1-2/OLE1* plants. The experiment was repeated 3 times with similar results. Bar = 50 μm. **b** TAG contents in mature leaves of *Col/OLE1*, *psat1-1/OLE1*, *psat1-2/OLE1* plants. Lipids were extracted from mature leaves of 4-week-old plants and TAGs were separated by TLC. Relative contents of TAG were quantified by Image J. Data are mean ± s.d. of four biological replicates. **c** Seed TAG contents of wild type and *psat* mutants quantified by GC-MS. Data are mean ± s.d. of three biological replicates. In **b** and **c,** different letters indicate significant differences at $P < 0.05$, as determined by one-way ANOVA with Tukey's multiple comparisons test.

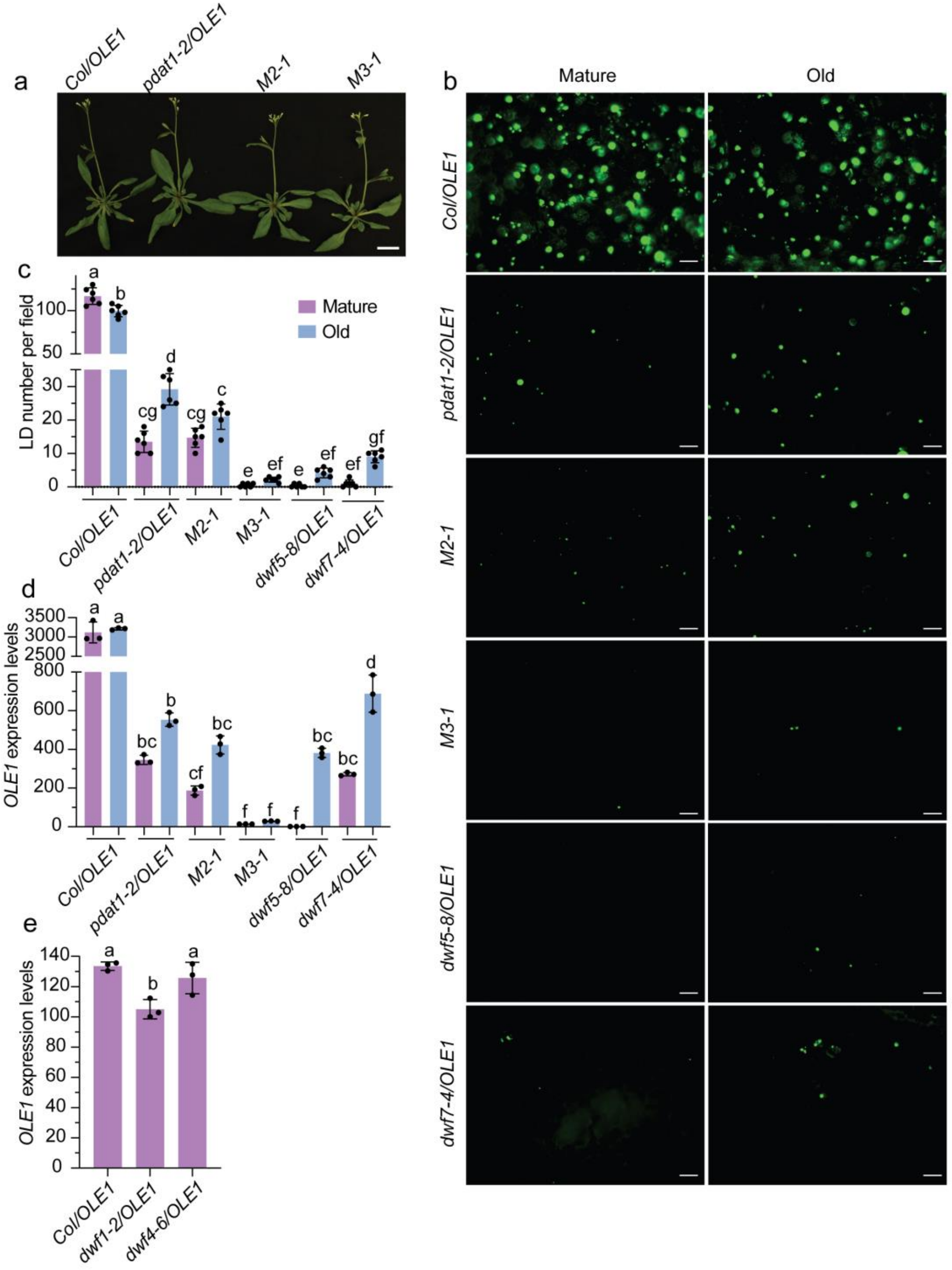

Supplementary Fig. 6 Expression levels of *OLE1* transgene are related to LD abundance. **a** Growth phenotype of 4-week-old wild type and LD-deficient mutants. Bar = 2 cm. **b** Representative images of LDs (OLE1-GFP, green) in leaves of 4-week-old *Col/OLE1* and LD-deficient mutant plants. Bar = 40 µm. The experiment was repeated 3 times with similar results. **c** LD number per microscopic field of mature and old (for definitions, see Fig. 1) leaves. LD numbers of the leaves were counted before RNA extraction and qRT-PCR analysis. Data are mean ± s.d. of six microscopic fields. **d** *OLE1* expression levels in mature and old leaves of 4-week-old *Col/OLE1* and LD-deficient mutant plants quantified by qRT-PCR. Data are mean ± s.d. of three biological replicates. **e** *OLE1* expression levels in mature leaves of 4-week-old *Col/OLE1*, *dwf1-2/OLE1* and *dwf4-6/OLE1* plants quantified by qRT-PCR. Data are mean ± s.d. of three biological replicates. In **c**, **d,** and **e**, different letters indicate significant differences at $P < 0.05$, as determined by one-way ANOVA with Tukey's multiple comparisons test.

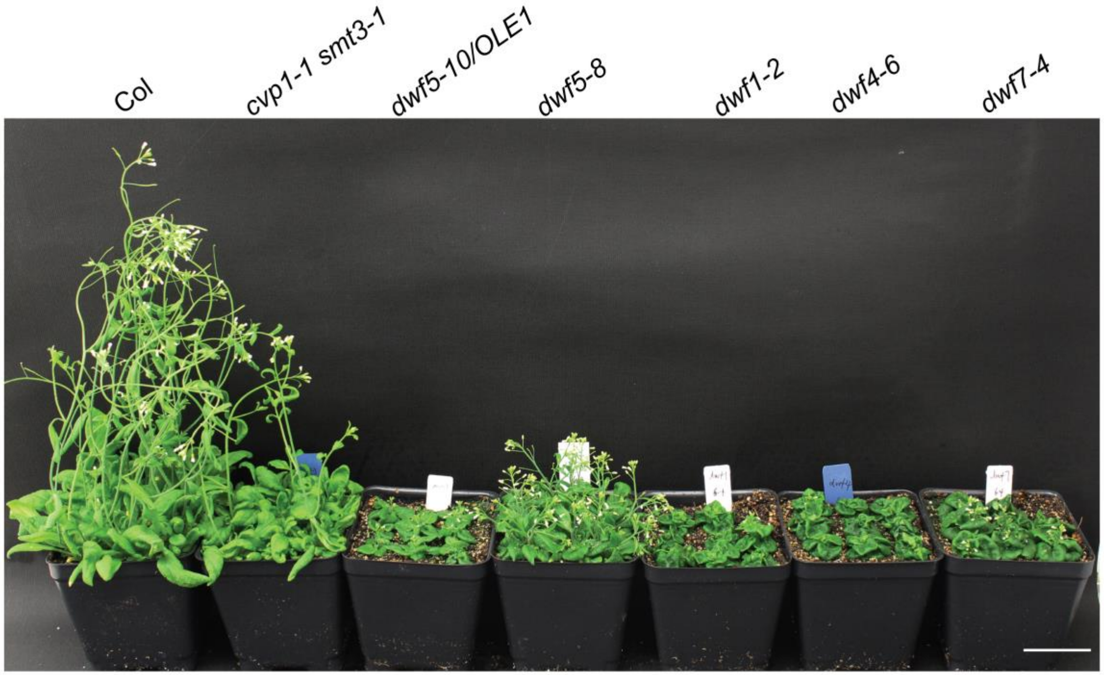

Supplementary Fig. 7 Five-week-old plants grown on soil. Bar=4 cm.

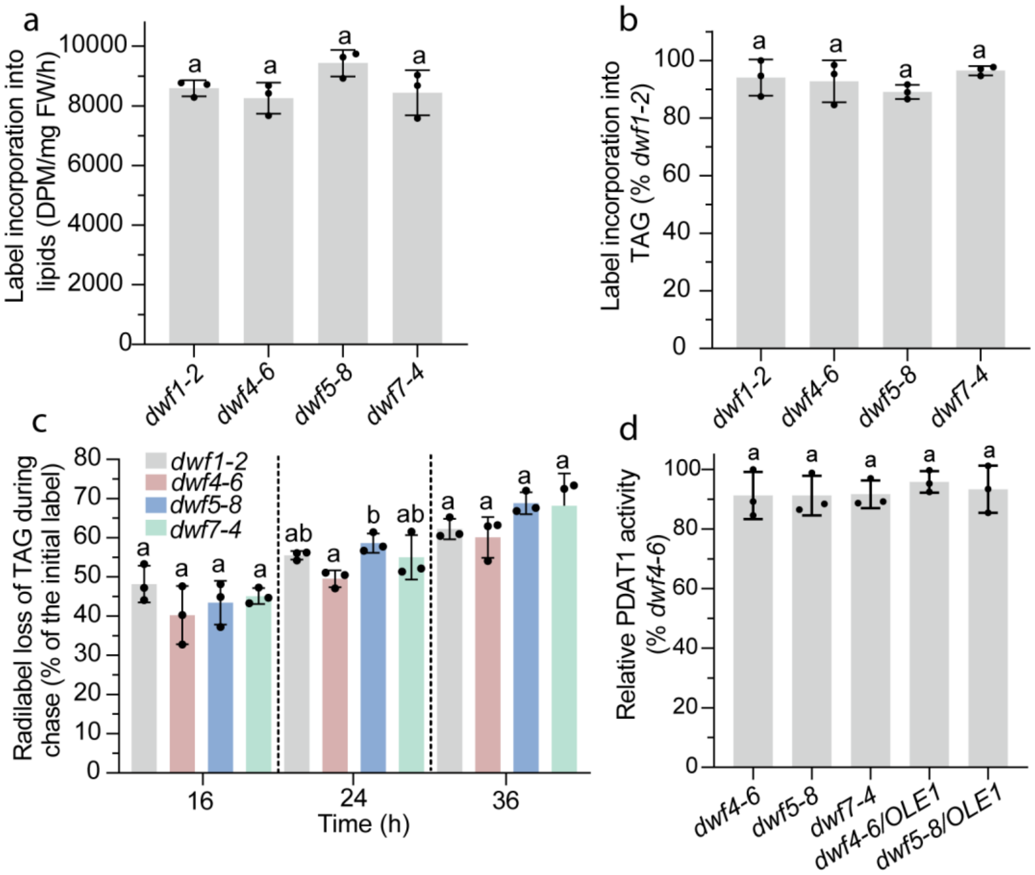

Supplementary Fig. 8 ^{14}C-acetate pulse-chase labeling assay and PDAT1 activity assay. a Initial rates of fatty acid synthesis in mature leaves of 4-week-old *dwf1-2*, *dwf4-6*, *dwf5-8* and *dwf7-4* measured by ^{14}C-acetate labeling for 1 h. Label incorporation into total lipids was measured by scintillation counting. **b** Label incorporation into TAG in *dwf1-2*, *dwf4-6*, *dwf5-8* and *dwf7-4* leaves by ^{14}C-acetate labeling for 1 h. **c** Radiolabel loss of TAG during the chase following 1 h incubation with ^{14}C-acetate. **d** Relative PDAT1 activity in microsomal membranes of mature leaves of *dwf4-6*, *dwf5-8*, *dwf7-4*, *dwf4-6/OLE1* and *dwf5-8/OLE1* plants. Data are mean ± s.d. of three biological replicates. Different letters indicate significant differences at $P < 0.05$, as determined by one-way ANOVA with Tukey's multiple comparisons test.

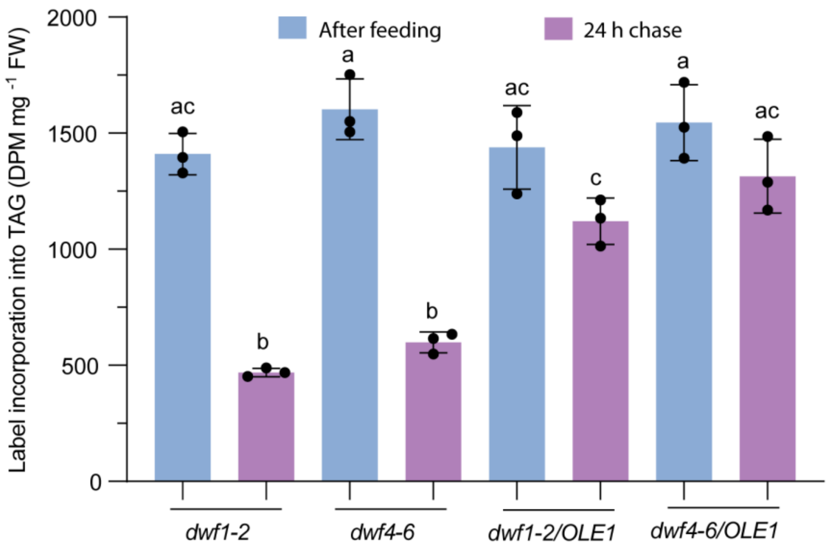

Supplementary Fig. 9 Label incorporation into TAG in leaves of *dwf1-2*, *dwf4-6*, *dwf1-2/OLE1*, and *dwf4-6/OLE1* after ^{14}C-oleic acid feeding and after 24 h of chase. Lipids were extracted from leaves and separated by TLC. Radiolabeled TAGs were quantified by liquid scintillation counting. Data are mean ± s.d. of three biological replicates. Different letters indicate significant differences at $P < 0.05$, as determined by one-way ANOVA with Tukey's multiple comparisons test.

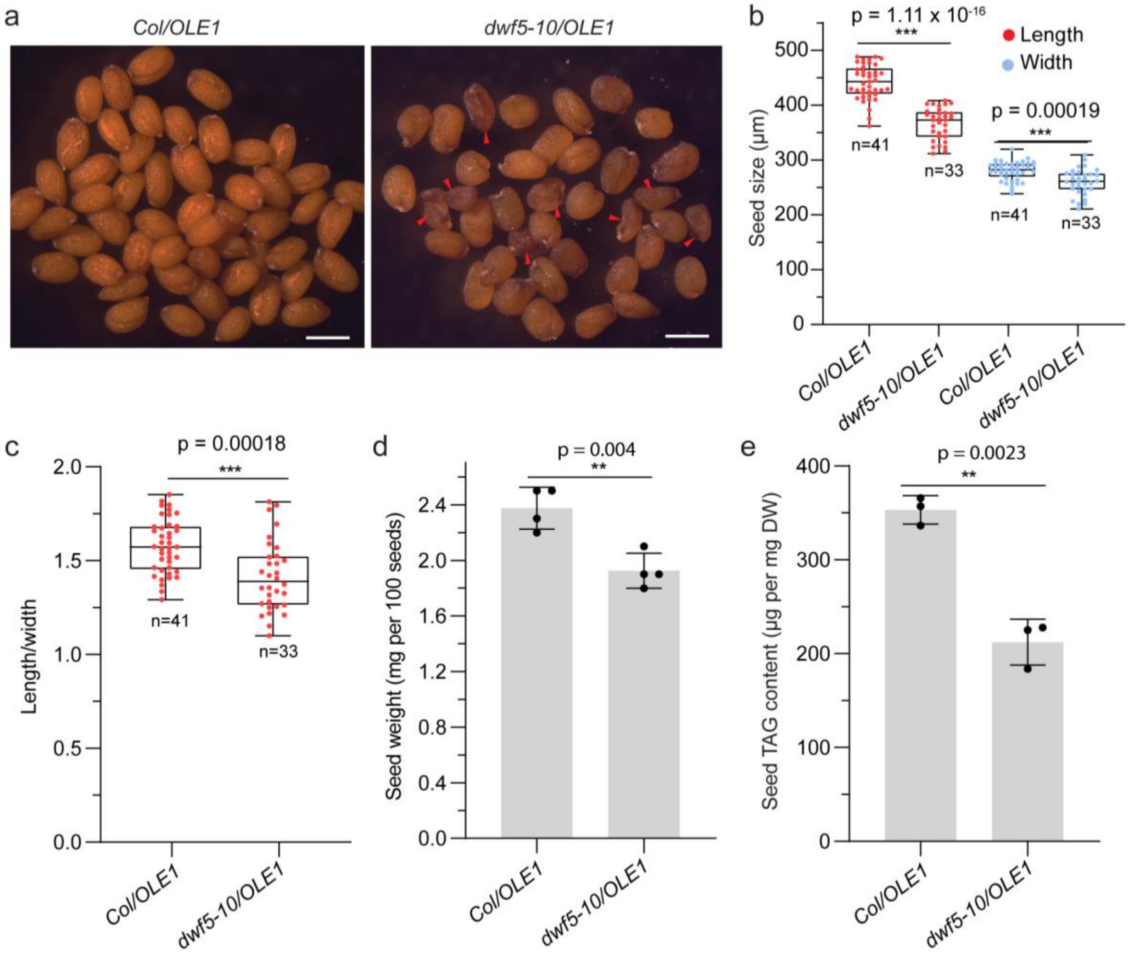

Supplementary Fig. 10 Seed morphology and TAG content altered in *dwf5-10/OLE1*. **a** Representative phenotype of dry seeds of *Col/OLE1* and *dwf5-10/OLE1*. Red triangles indicate severe wrinkled seeds. Bar = 400 µm. **b** Seed size. **c** Length to width ratio of seeds. **d** Weight per 100 seeds. Data are mean ± s.d. of four biological replicates. **e** Seed TAG contents. Data are mean ± s.d. of three biological replicates. In **b** and **c**, n represents the number of seed used for data collection. The box plot boundaries reflect the interquartile range, the centre line is the median and the whiskers represent 1.5× the interquartile range from the lower and upper quartiles. In **b**, **c**, **d,** and **e**, asterisks indicate statistically significant differences based on a two-sided Student's *t* test (**P* < 0.05, ***P*< 0.01, ****P*< 0.001).

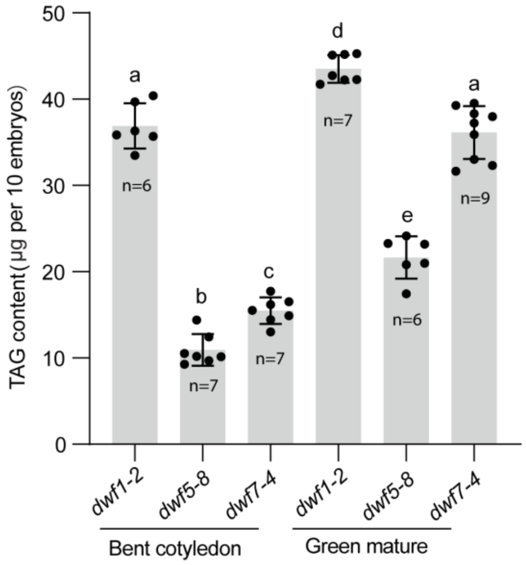

Supplementary Fig. 11 TAG contents in *dwf1-2, dwf5-8, dwf7-4* embryos at bent cotyledon and green mature stage. Embryos were dissected under dissecting microscope, and 10 embryos at bent cotyledon or mature green stage were used for TAG analysis by GC-MS. n represents the number of biological replicates. Data are mean ± s.d.. Different letters indicate significant differences at $P < 0.05$, as determined by one-way ANOVA with Tukey's multiple comparisons test.

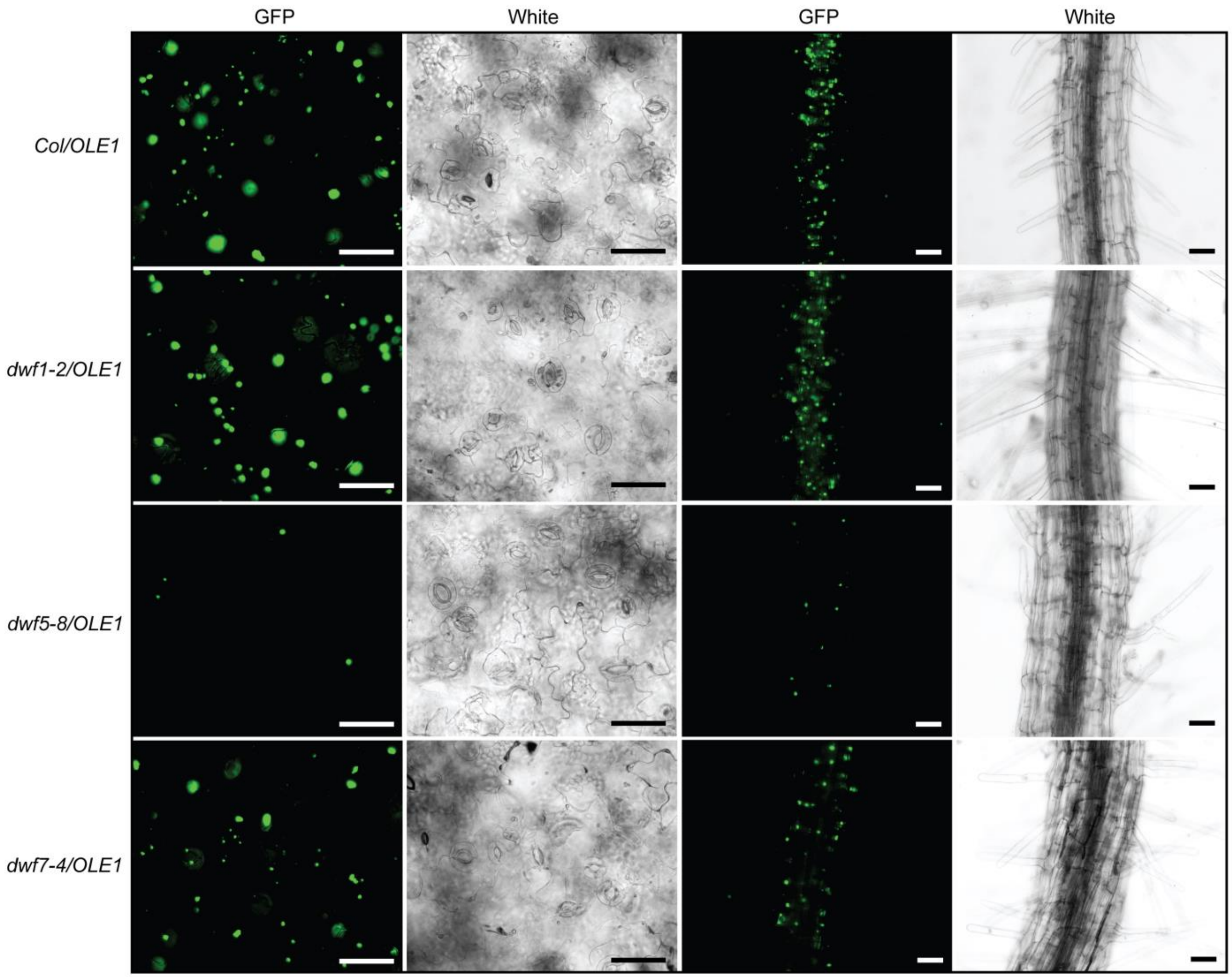

Supplementary Fig. 12 LDs in root and cotyledon of young seedlings. LDs (OLE1-GFP, green) in root and cotyledon of 6-day-old seedlings were visualized under a Zeiss epifluorescence microscope. The experiment was repeated 3 times with similar results. Bar = 50 μm.

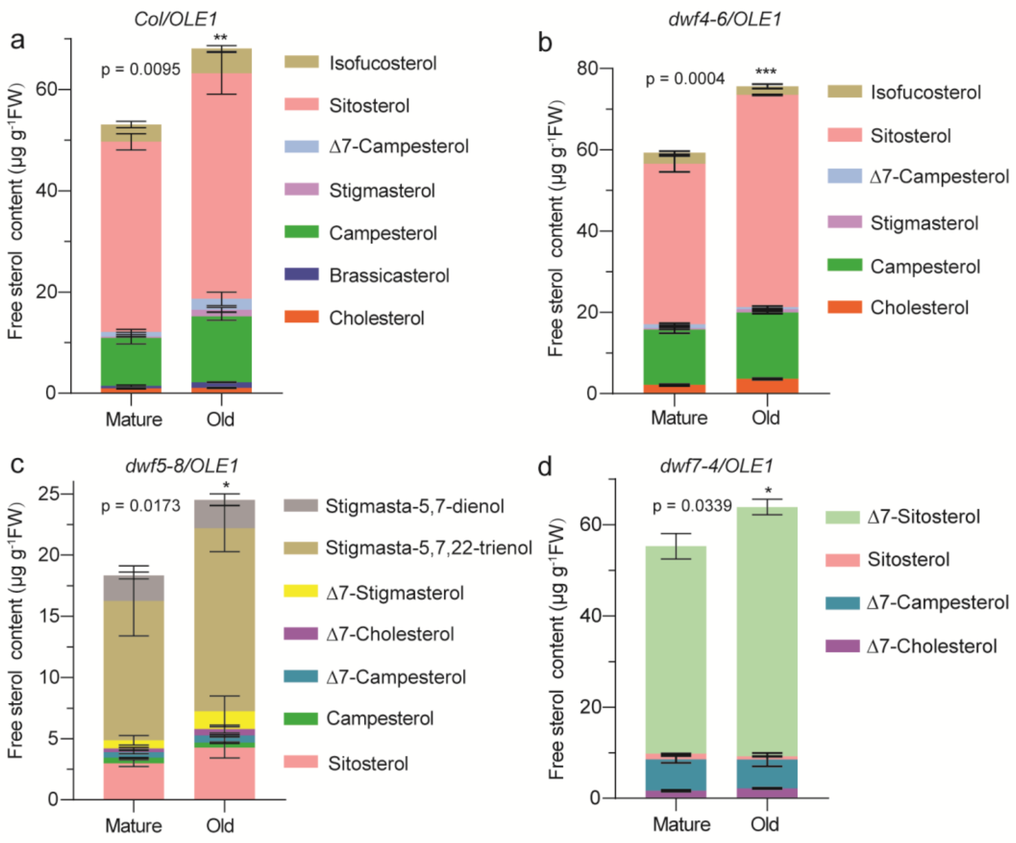

Supplementary Fig. 13 Free sterol levels in mature and old leaves. Free sterol contents of mature and old leaves of 4-week-old *Col/OLE1*, *dwf4-6/OLE1*, *dwf5-8/OLE1* and *dwf7-4/OLE1* plants were measured by GC-MS. Data are mean ± s.d. of three biological replicates. Asterisks indicate significant differences, as determined by a two-sided Student's *t* test (*$P < 0.05$, **$P < 0.01$, ***$P < 0.001$).

Supplementary Table 1 Sterol composition in mature leaves of 4-week-old wild type, *dwf* and *psat* mutants. Values are given in µg per g fresh weight. Data are mean ± s.d. of three biological replicates.

Sterols	Genotype									
	Col	*dwf1-2*	*dwf4-6*	*dwf5-8*	*dwf7-4*	*cvp1-3 smt3-1*	*psat1-1*	*psat1-2*	*Col/OLE1*	*dwf5-10/OLE1*
Cholesterol	0.93 ±0.27		1.15±0.12	0.24±0.07		0.96±0.34	0.62±0.12	1.25±0.22	1.02±0.03	0.45±0.22
Brassicasterol	0.63±0.20					1.19±0.37		0.68±0.11	0.71±0.54	
Δ7-Cholesterol										0.80±0.12
24-Methylenecholesterol		7.49 ±0.66				8.84±0.67			1.82±1.31	
Campesterol	6.89 ±0.84		8.97±0.96	0.57±0.16	0.12±0.10	33.00±10.33	6.92±0.27	10.18±2.58	8.96±1.17	0.57±0.21
Stigmasterol	0.32 ±0.05	0.17 ±0.07	0.26±0.10			0.92±0.45	0.67±0.19	0.39±0.08	0.86±0.19	
Δ7-Campesterol	5.36 ±0.92		5.89±0.76	0.70±0.29	1.94±0.01	1.83±1.44	12.87±1.43	8.88±0.67	10.20±0.88	0.65±0.09
Sitosterol	29.45 ±2.37	1.20 ±0.11	27.20±3.14	2.22±1.02	0.30±0.02	10.94±3.48	27.10 ±3.44	40.72±6.85	32.34±3.82	
Δ7-Stigmasterol				1.48±1.24						1.43±0.22
Isofucosterol	2.56 ±0.25	44.93 ±3.48	2.54±0.61			2.70±0.73	2.25 ±0.40	4.74±1.15	4.80±0.49	0.78±0.10
Stigmasta-5,7,22-trienol				6.23±0.77						8.19±1.60
Stigmasta-5,7-dienol				1.26±0.48						3.07±0.22
Δ7-Sitosterol					34.92±1.43					
Total	46.14 ±3.30	53.34 ±3.94	45.99±5.59	12.70±3.16	37.24±1.36	55.71±8.35	49.3 ±3.64	66.84±9.15	60.70±5.56	15.66±0.90

Supplementary Table 2 Sterol-ester composition in mature leaves of 4-week-old wild type, *dwf* and *psat* mutants. Values are given in µg per g fresh weight. Data are mean ± s.d. of three biological replicates.

	Genotype									
Sterols	col	*dwf1-2*	*dwf4-6*	*dwf5-8*	*dwf7-4*	*cvp1-3 smt3-1*	*psat1-1*	*psat1-2*	*Col/OLE1*	*dwf5-10/OLE1*
Cholesterol	0.32±0.11		0.33±0.06				0.27±0.07	0.10±0.09	0.39±0.06	
Brassicasterol									0.71±0.54	
24-Methylenecholesterol		2.12±0.14								
Campesterol	3.93±0.61		2.73±0.15		0.13±0.09	0.73±0.39	2.01±0.53	0.53±0.07	2.35±1.87	1.48±0.34
Stigmasterol	0.28±0.10									
Δ7-Campesterol	0.92±0.30		0.48±0.21		0.14±0.02		0.43±0.07		0.66±0.16	
Δ5,22,25-Stigmasterol		0.79±0.18								
Sitosterol	15.48±4.02	0.34±0.08	7.70±0.76	0.57±0.17		0.57±0.12	2.18±0.45	0.68±0.19	15.93±2.22	0.57±0.04
Δ7-Stigmasterol				0.61±0.23		0.39±0.03				
Isofucosterol	3.44±1.45	9.07±0.61	1.01±0.12				0.17±0.02	0.13±0.12	4.90±3.08	
Stigmasta-5,7,22-trienol				2.29±0.82		2.27±0.52				1.98±0.14
Stigmasta-5,7-dienol				0.64±0.41		1.02±0.29				0.65±0.22
Δ7-Sitosterol					2.18±0.08					
Total	23.76±6.76	12.33±0.53	12.24±0.95	4.12±1.13	2.46±0.02	4.98±1.33	5.05±1.10	1.44±0.33	23.85±5.20	4.96±0.56

Supplementary Table 3 Sterol composition in dry seeds of wild type, *dwf* and *psat* mutants.
Values are given in µg per g dry weight. Data are mean ± s.d. of three biological replicates.

Sterols	Genotype								
	Col	*dwf1-2*	*dwf4-6*	*dwf5-8*	*dwf7-4*	*cvp1-3 smt3-1*	*psat1-1*	*Col/OLE1*	*dwf5-10/OLE1*
Cholesterol	5.54±0.64		3.35±0.51			12.79±3.49	4.46±0.62	8.39±6.89	5.72±1.74
Brassicasterol	10.15±1.61					12.81±0.08	5.66±0.30	6.52±1.63	
24-methylenecholesterol		27.50±3.68				130.91±26.57	31.33±8.19	17.93±4.81	
Campesterol	50.72±15.56		66.01±19.56	8.62±2.59	20.64±2.43	330.60±88.50	71.80±16.95	52.64±3.03	5.83±0.44
Stigmasterol	27.52±8.77		8.97±2.37				10.53±7.92	4.33±3.45	
Δ7-Campesterol	62.44±14.87	7.68±1.95	76.55±3.35	15.25±6.61	41.60±7.73		144.54±19.72	95.53±19.46	13.61±3.82
Δ5,22,25-Stigmasterol		101.52±29.85							
Sitosterol	400.60±53.96	29.04±8.77	327.37±47.94	17.26±1.90	41.74±8.70	6.90±1.57	380.75±65.90	287.91±25.74	17.70±5.80
Δ7-Stigmasterol				59.62±7.63					45.67±7.22
Isofucosterol	33.74±12.45	328.01±85.82	27.85±5.18				11.29±6.81	20.27±4.07	
Stigmasta-5,7,22-trienol				36.58±8.09					18.32±3.28
Stigmasta-5,7-dienol				27.29±4.29					19.75±3.69
Δ7-Sitosterol					153.30±24.83				
Total	590.70±62.47	493.75±91.82	510.09±65.95	164.61±19.74	257.29±21.11	497.74±79.02	661.08±83.55	493.22±30.91	124.70±21.22

Supplementary Table 4 Primers used in this study.

Primers used for mapping						
			Size of PCR product			
Marker name	Location in genome	Type	Col	Ler	Forward primer sequences	Reverse primer sequences
1-5886	17911k	sslp	141	124	TCCGATCTTCACTATTATTT	ACCTAATATCGGATCAATTC
1-6115	18605k	sslp	144	126	CCACTAGACAAGTACAAGAT	CACAAGAAAACCCTGTATTA
1-6395	19459k	sslp	173	148	GTCTTGGAAACTACATTTGTTCAT	GTTCCAATGATAGTGAATTGTGAG
1-6556	19947k	sslp	126	96	TAAAGATACGATTCGTAAGA	CTAGCAGGCAAATGAGAC
nga280	20873k	sslp	105	85	CTGATCTCACGGACAATAGTGC	GGCTCCATAAAAAGTGCACC
1-7199	21906k	sslp	152	207	AGATGCAAATTCCAAGAAAC	GCCAGACATACTTTCATGTG

Primers used for qRT-PCR	
DWF5 qRT-PCR FP	ACATTGCACATGACCGAGCTG
DWF5 qRT-PCR RP	AAGGCACCCACACTAGACAACC
UBQ5 qRT-PCR FP	AGAAGATCAAGCACAAGCAT
UBQ5 qRT-PCR RP	CAGATCAAGCTTCAACTCCT
OLE1 qRT-PCR FP	AGGCAGATTGCTAAAGCTGCAAC
OLE1 qRT-PCR RP	ACTGTGATGAGAGCCGGG
