# Supplementary material for: Sterols are required for the coordinated assembly of lipid droplets in developing seeds
Source: Nat Commun. 2021 Sep 22;12:5598. doi: 10.1038/s41467-021-25908-6 (PMC8458542; doi:10.1038/s41467-021-25908-6)
Supplement: Supplementary file 3 — Description of Additional Supplementary Files [file 41467_2021_25908_MOESM3_ESM.pdf]

Description of Additional Supplementary Files

Supplementary Data 1: Mutations identified in the region between the marker 1-5886 and nga280 by Mutmap sequencing
